# Screening and identification of lncRNAs as potential biomarkers for pulmonary tuberculosis

**DOI:** 10.1038/s41598-017-17146-y

**Published:** 2017-12-01

**Authors:** Zhong-liang Chen, Li-Liang Wei, Li-Ying Shi, Meng Li, Ting-Ting Jiang, Jing Chen, Chang-Ming Liu, Su Yang, Hui-hui Tu, Yu-ting Hu, Lin Gan, Lian-Gen Mao, Chong Wang, Ji-Cheng Li

**Affiliations:** 1 Institute of Cell Biology, Zhejiang University, Hangzhou, 310058 P.R. China; 2 Department of Respiratory Medicine, The Sixth Hospital of Shaoxing, Shaoxing, 312000 P.R. China; 3 Department of Clinical Laboratory, Zhejiang Hospital, Hangzhou, 310013 P.R. China; 4 South China University of Technology School of Medicine, Guangzhou, 510006 P.R. China

## Abstract

Pulmonary tuberculosis (TB) is among the diseases with the highest morbidity and mortality worldwide. Effective diagnostic methods for TB are lacking. In this study, we investigated long non-coding RNAs (lncRNAs) in plasma using microarray and the potential diagnostic value of lncRNAs for TB. We found a total of 163 up-regulated lncRNAs and 348 down-regulated lncRNAs. Gene ontology (GO), Kyoto Encyclopedia of Genes and Genomes (KEGG) and coding-noncoding co-expression (CNC) analyses showed that functions of differentially expressed lncRNAs were mainly enriched in the regulation of alpha-beta T cell activation and the T cell receptor signalling pathway. Four differentially expressed lncRNAs, NR_038221 (fold change = 3.79, P < 0.01), NR_003142 (fold change = 1.69, P < 0.05), ENST00000570366 (fold change = 3.04, P < 0.05), and ENST00000422183 (fold change = 2.11, P < 0.001), were verified using RT-qPCR. Among those, NR_038221, NR_003142, and ENST00000570366 were found to be up-regulated, while ENST00000422183 was down-regulated. The value of the area under the curve (AUC) for the diagnostic model consisting of the four lncRNAs was 0.845 (sensitivity = 79.2%, specificity = 75%). We further predicted 85 mRNAs and 404 miRNAs that potentially interact with these lncRNAs. Our study revealed the potential value of lncRNAs as biomarkers for early diagnosis of TB and the underlying mechanisms of these abnormally expressed lncRNAs in the pathogenesis of TB.

## Introduction

Tuberculosis (TB) is a chronic pulmonary infection caused by *Mycobacterium tuberculosis* (*MTB*). It is one of the ten diseases with the highest mortalities in the world. According to the World Health Organization (WHO) report, there were an estimated 10.4 million new cases of TB and 1.4 million deaths due to TB in 2015 worldwide. China ranks third among the high TB burden countries, with 0.92 million new cases of TB in 2015, accounting for approximately 9% of the global incidence, and the death toll was approximately 35 thousand. China is also one of the five countries with the greatest burden of multidrug resistant (MDR)-TB^1^.

Accurate and early diagnosis is important for controlling infection and effective treatment of TB^2^. In China, TB diagnosis is made by clinical manifestations, radiological and pathological changes, and acid-fast bacilli (AFB) culture for *Mycobacterium*. However, the detection rate of AFB in newly infected TB patients is only 44% (15–20% in children)^3^. The detection of *MTB* is the gold standard for diagnosis of TB. However, *MTB* culture takes 3–4 weeks, which may not be conducive to controlling the infection^4^. Although interferon gamma release assays (IGRA) have been used to diagnose TB, great differences between individuals have suggested that they are not suitable for TB diagnosis^5^. Therefore, effective methods for early diagnosis are imperative for preventing TB. Our previous studies demonstrated the potential value of miRNAs and several proteins as biomarkers for early diagnosis of TB, and we also reported biomarkers for cured TB^6–9^.

In recent years, long non-coding RNAs (lncRNAs) have been shown to play important roles in many diseases and have been demonstrated to be associated with many physiological and pathological processes, such as embryonic development, immune responses, cardiovascular diseases and cancer^10–14^. Meanwhile, studies have also shown that lncRNAs can serve as molecular biomarkers in the diagnosis and prognosis of many diseases, especially in tumours^15,16^. Abnormal expression of lncRNAs has been demonstrated in CD8+ T cells, CD4+ T cells, B lymphocytes, and macrophages in patients with TB, suggesting that lncRNAs are closely related to the pathological processes of TB^17–21^.

In this study, we used lncRNA microarrays to explore potential diagnostic biomarkers in the plasma of patients with TB and studied the interactions between lncRNAs, miRNAs and mRNAs. Our study provides a new basis for early diagnosis of TB and reveals the potential pathogenesis of TB.

## Results

### Patient characteristics

There were no significant differences between the TB patient group and the healthy control group in gender and age (P > 0.05) (Table 1). The data showed abnormal expression levels of proteins in TB patients, compared with normal reference ranges. The expression levels of IgG (P = 0.006), IgA (P = 0.001), IgM (P < 0.001), and C reactive protein (P = 0.003) were significantly higher in TB patients, while the expression level of complement 3 (C3) was lower than the normal reference range (Table 2).

**Table 1:** Characteristics of pulmonary TB patients and healthy control subjects.

		Pulmonary TB	Healthy control	P value
Microarray	Age	38.55 ± 15.68	36.33 ± 7.99	0.82^a^
	Gender (male)	33 (19)	11(7)	0. 72^b^
qPCR verification	Age	41.35 ± 17.27	38.92 ± 10.60	0.80^a^
	Gender (male)	52 (44)	52 (40)	0.31^b^

A P-value ≤ 0.05 indicates statistical significance. ^a^P value between TB patients and healthy control subjects, t test; ^b^P value between Pulmonary TB patients and controls, χ^2^ test.

**Table 2:** Clinical data of pulmonary TB patients (N = 187) and normal reference ranges.

	TB patients	Normal reference ranges	Median	P value
Total protein (g/L)	70.60 ± 6.89	64.00–83.00	72.88	0.004^**^
Albumin (g/L)	40.34 ± 6.62	36.00–52.00	43.27	<0.001^***^
Globulin (g/L)	30.2723 ± 5.62	22.00–34.00	27.35	<0.001^***^
A/G	1.38 ± 0.34	1.20–2.50	1.73	<0.001^***^
CRP (mg/L)	25.39 ± 40.56	0.00–0.82	0.41	<0.001^***^
Prealbumin (g/L)	0.17 ± 0.10	0.15–0.36	0.23	<0.001^***^
IgG (g/L)	14.40 ± 5.16	11.50–14.22	12.79	0.006^**^
IgA (g/L)	2.84 ± 1.33	1.70–3.25	2.35	0.001^***^
IgM (g/L)	1.40 ± 0.79	0.73–1.17	0.92	<0.001^***^
Complement C3	1.14 ± 0.21	0.83–1.77	1.21	0.003^**^
Complement 4 (mg/L)	314 ± 101.12	100.00–400.00	200	<0.001^***^

A P-value ≤ 0.05 indicates statistical significance. ^**^P < 0.01; ^***^P < 0.001. P value between TB patients and normal reference range, for one-sample t-test after taking the logarithm then compare to the median.

### Differentially expressed lncRNAs and mRNAs in TB patients

**Figure 1:**
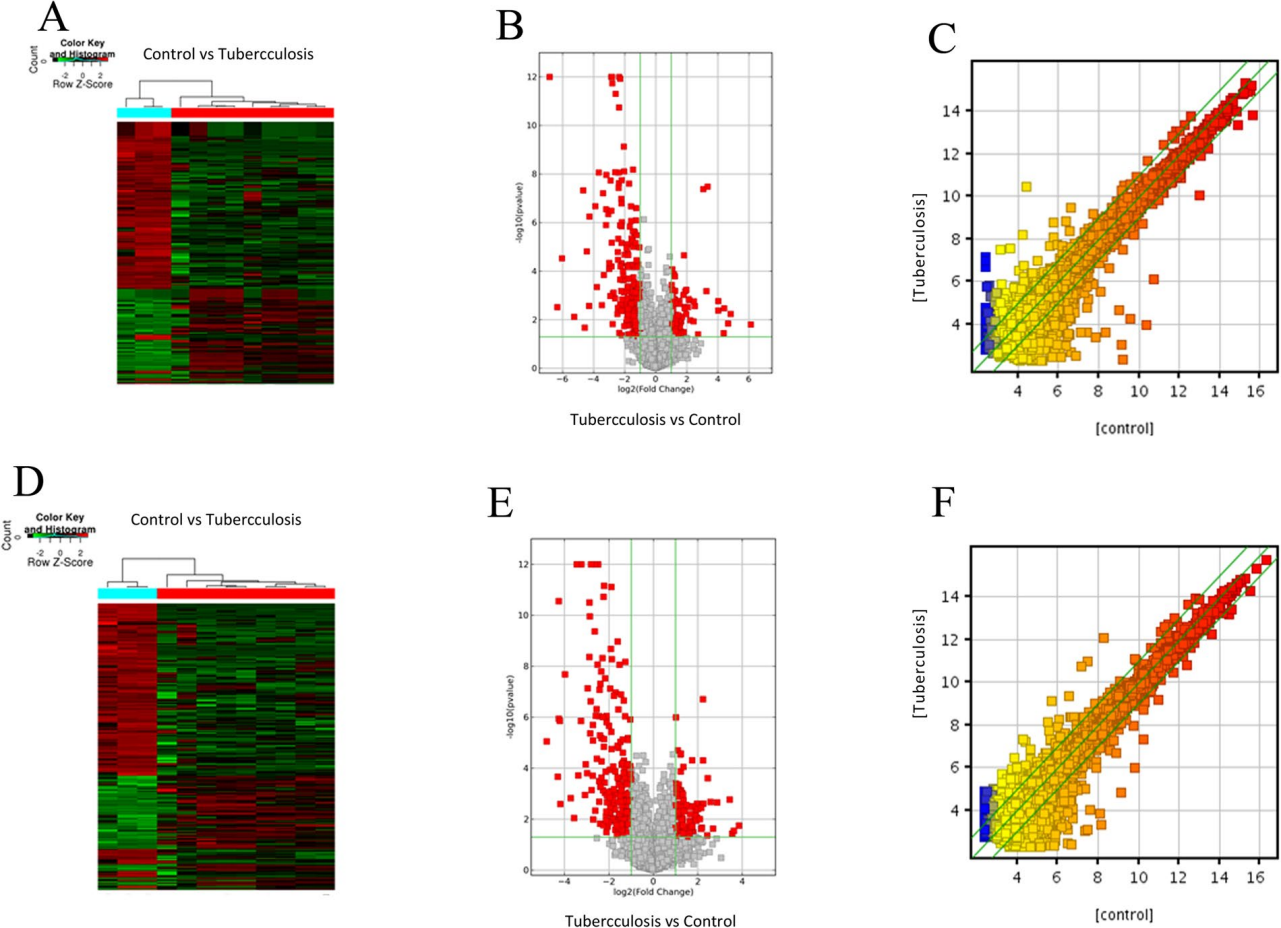
Differentially expressed lncRNAs and mRNAs between TB patients and healthy control subjects. (**A**–**C**) The Hierarchical Clustering Map, Scatter Plot and Volcano Plot of the differentially expressed lncRNAs, respectively. (**D**–**F**) The Hierarchical Clustering Map, Scatter Plot and Volcano Plot of the differentially expressed mRNAs, respectively.

An Arraystar lncRNAs microarray was used to identify abnormally expressed lncRNAs and mRNAs in the plasma of newly diagnosed TB patients and healthy controls. LncRNAs and mRNAs were considered differentially expressed if the fold changes were >2.00 and the P value was <0.05. A total of 511 differentially expressed lncRNAs (163 up-regulated, 348 down-regulated) and 411 differentially expressed mRNAs (127 up-regulated, 284 down-regulated) were detected between TB patients and healthy controls. The Volcano plots, scatter plots, and cluster maps of differentially expressed lncRNAs and mRNAs are shown in Fig. 1.

### GO and KEGG analysis

**Figure 2:**
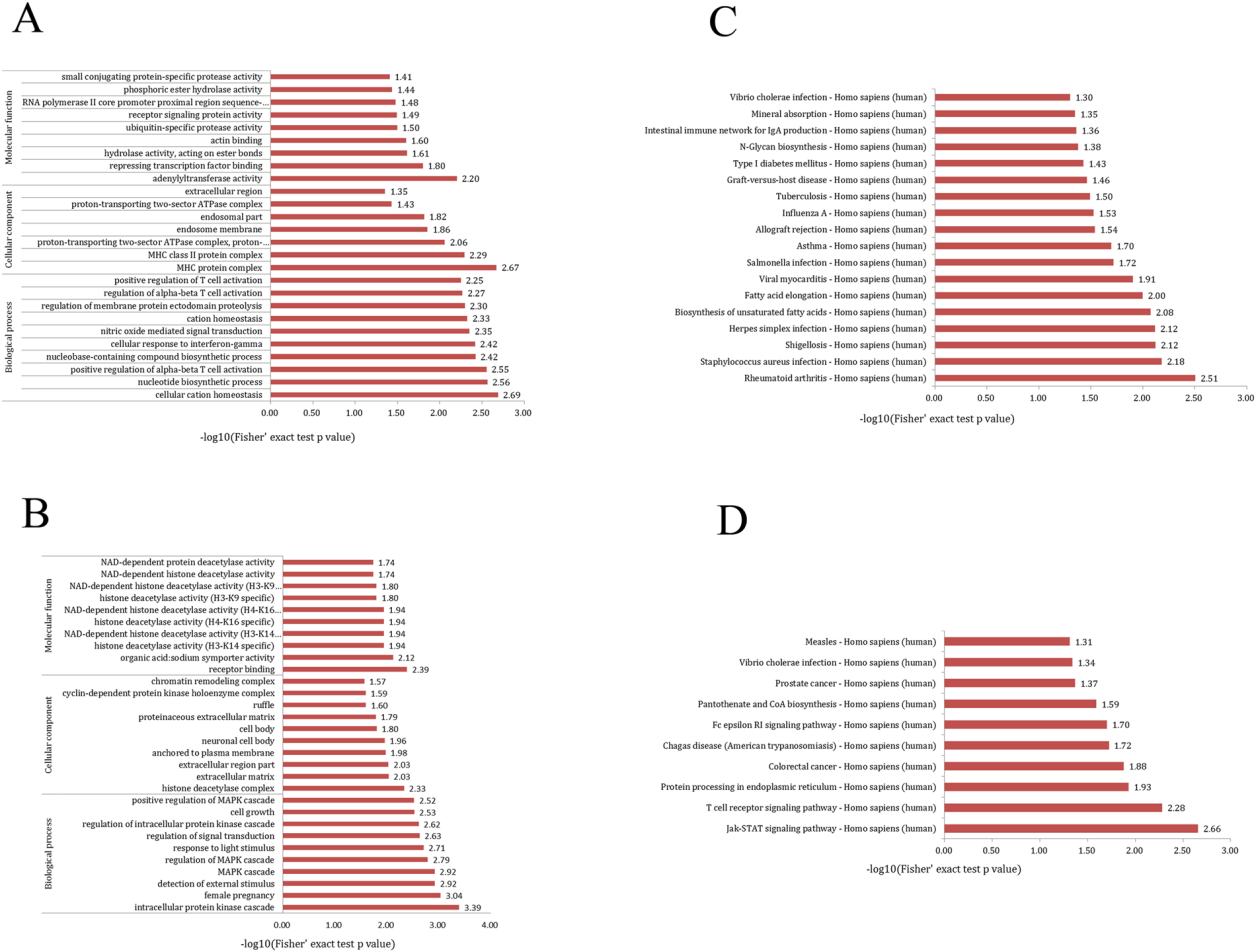
GO and KEGG pathway analysis of differentially expressed mRNAs between TB patients and healthy control subjects. The most enriched GO items of the up-regulated mRNAs (**A**) and down-regulated mRNAs (**B**) according to biological process (BP), cellular components (CC) and molecular functions (MF). The most enriched items of the KEGG pathway analysis of the up-regulated mRNAs (**C**) and down-regulated mRNAs (**D**).

A GO analysis of differentially expressed mRNAs was performed to predict potential gene functions with categories of biological processes (BP), cellular components (CC) and molecular functions (MF). The enriched GO terms associated with up-regulated mRNAs in TB patients included positive regulation of alpha-beta T cell activation, cellular response to interferon-gamma (IFN-γ), positive regulation of T cell activation, and the major histocompatibility (MHC) protein complex. The enriched GO terms associated with down-regulated mRNAs in TB patients included negative selection of T cells, detection of external stimulus, the intracellular protein kinase cascade, and the mitogen-activated protein kinase (MAPK) cascade. The GO results are shown in Fig. 2.

The KEGG pathways terms associated with up-regulated mRNAs in TB patients were *Staphylococcus aureus*, *Shigellosis*, *Herpes simplex*, and *Mycobacterium tuberculosis* infection. The KEGG pathway terms associated with down-regulated mRNAs in TB patients were Jak-STAT signalling, T cell receptor signalling, protein processing in the endoplasmic reticulum, and *Vibrio cholerae* infection pathways. The KEGG Pathway results are shown in Fig. 2.

### Coding non-coding co-expression (CNC) network and the selection of lncRNAs

**Figure 3:**
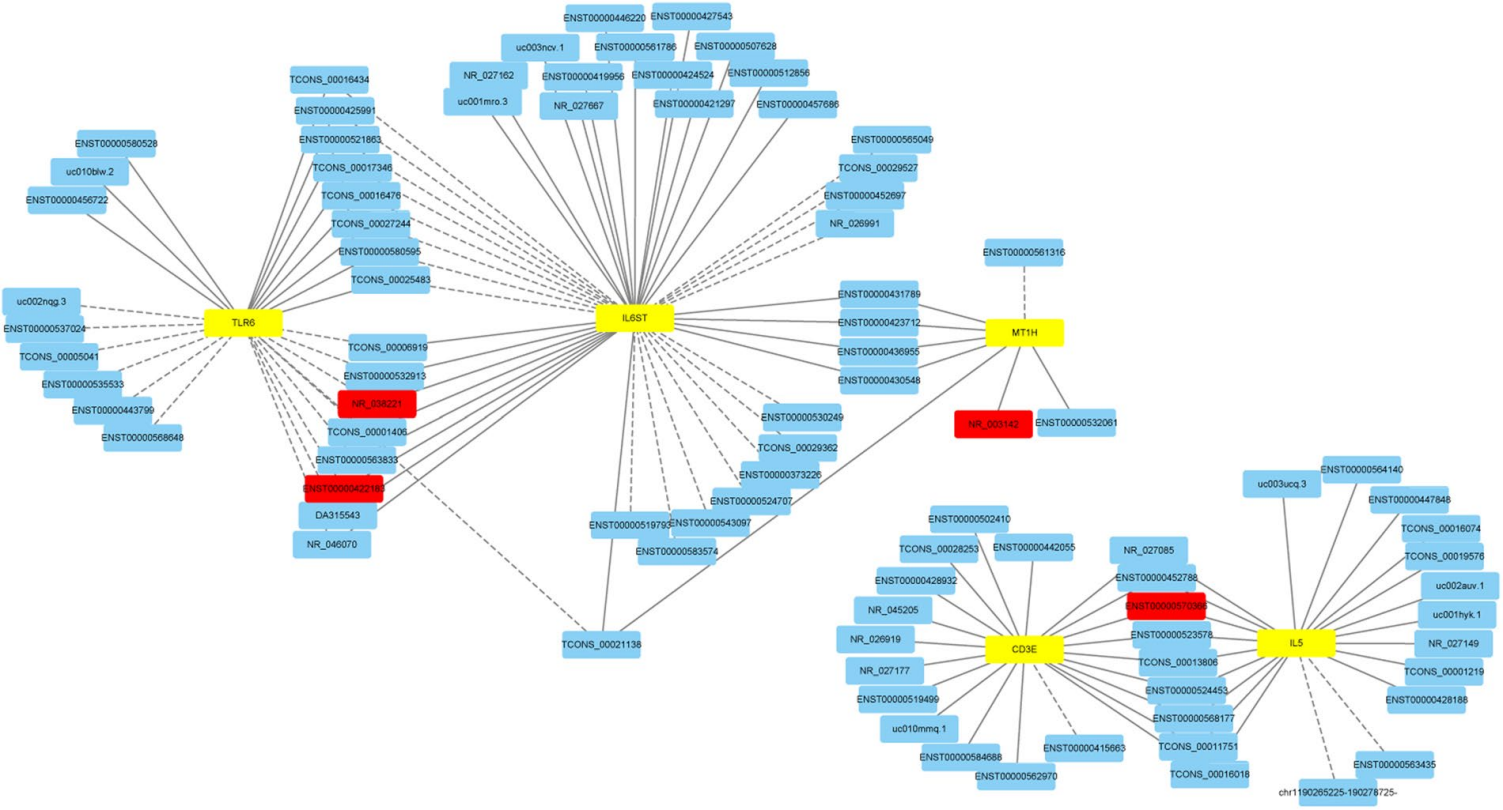
Part results of the CNC co-expression network. The yellow node represents the coding gene, the blue node represents lncRNAs, and the red node represents the verified lncRNAs. The solid line between the two nodes represents a positive correlation, and the dotted line represents a negative correlation. This figure showed that NR_038221 and ENST00000422183 had positive correlations with IL6ST and negative correlations with TLR6, NR_003142 had positive correlation with MT1H, and ENST00000570366 had positive correlations with CD3E and IL5.

According to the GO and KEGG analysis results of the differentially expressed mRNAs, GO and KEGG pathways that might be related to the disease progression of TB were selected and classified into three categories: (i) Potentially associated with the regulation and differentiation of T cell; (ii) Antigen presentation and transmembrane signal transduction; and (iii) The intracellular signal transduction pathway. The precise GO and KEGG pathways of these three categories are shown in Supplementary Table 1. The CNC network was constructed based on these three categories. LncRNAs associated with IL6ST, IL18, BCL2, IL5, FYN, MAPK9 and CD3E were in category (i), lncRNAs associated with TLR6, NOD2, TIMP1 and HLA-DQB1 were in category (ii), and lncRNAs associated with MAPK9, MKNK2, CC2D1A, IL18, TLR6, FYN and NOD2 were in category (iii). The complete results of the CNC network are shown in Supplementary Figure S1. LncRNAs associated with genes included in the categories described above with a normalized intensity > = 5.0 in the microarray results were selected, and a total of 30 lncRNAs were selected. Detailed information of those lncRNAs is shown in Supplementary Table 2. Further CNC analysis of the verified differentially expressed lncRNAs revealed that NR_038221 and ENST00000422183 had a positive correlation with IL6ST and a negative correlation with TLR6, NR_003142 had a positive correlation with MT1H, and ENST00000570366 had a positive correlation with CD3E and IL5. The CNC analysis associated with the verified lncRNAs is shown in Fig. 3.

### RT-qPCR identification of the candidate lncRNAs

**Figure 4:**
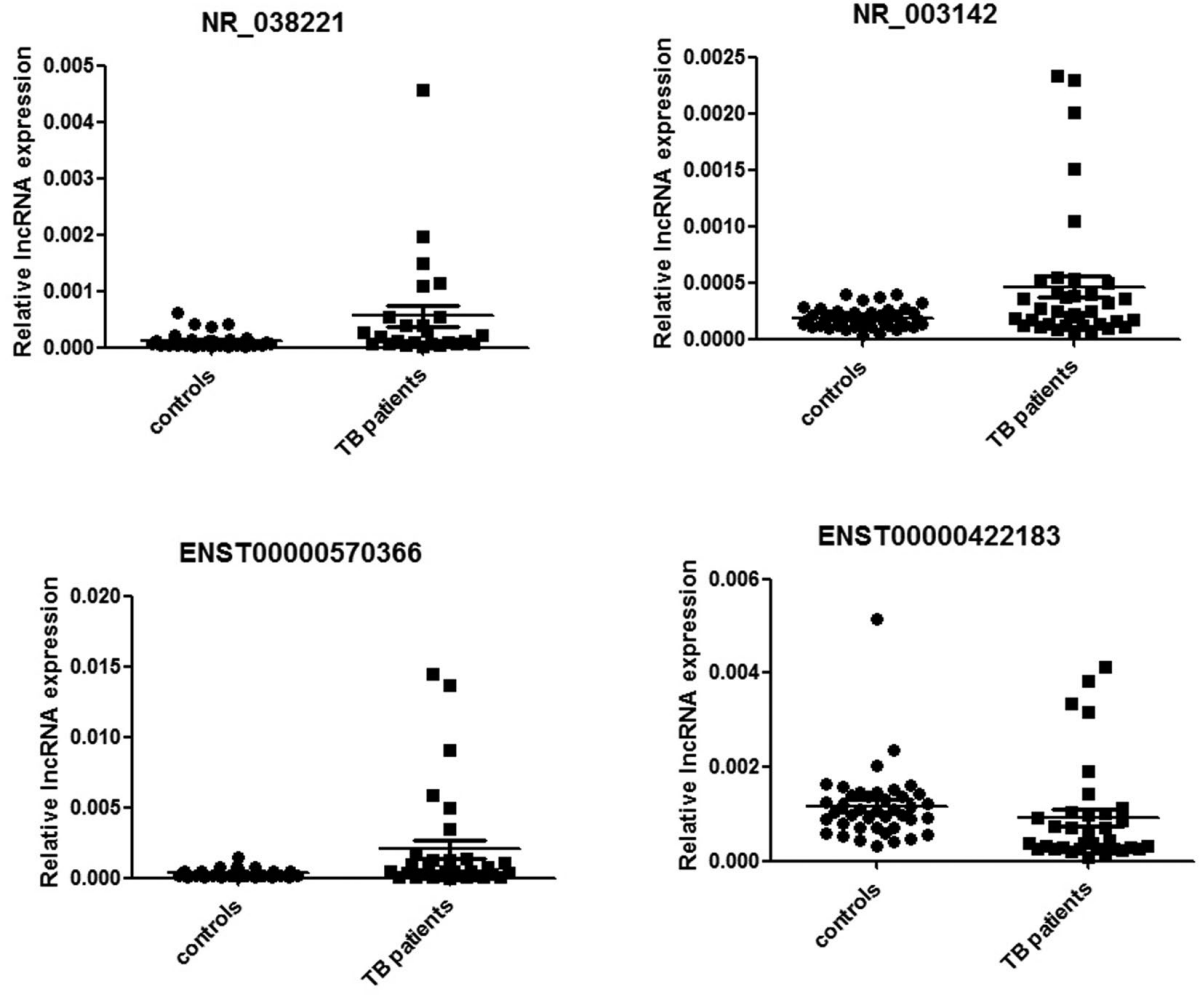
The qRT-PCR validation of lncRNA expression levels in TB patients and healthy control subjects. Three lncRNAs were up-regulated in TB patients: NR_038221 (fold change = 3.79, P < 0.01), NR_003142 (fold change = 1.69, P < 0.05), and ENST00000570366 (fold change = 3.04, P < 0.05). ENST00000422183 (fold change = 2.11, P < 0.001) was down-regulated in TB patients compared with the healthy control subjects.

All lncRNAs listed in Supplementary Table 2 were identified using the methods described below in 15 TB patients and 15 healthy control subjects. LncRNAs that met the following conditions were excluded from further verification: (i) Non-specific amplification and primer dimerization (ii) and LncRNAs with a low copy number that were hard to detect using the qPCR method (Ct value > 35). Finally, six lncRNAs, NR_038221, NR_003142, ENST00000568177, ENST00000570366, ENST00000422183, and ENST00000449589, were selected and verified in 52 TB patients and healthy control subjects. The qPCR results showed that the expression levels of NR_038221 (fold change = 3.79, P < 0.01), NR_003142 (fold change = 1.69, P < 0.05), and ENST00000570366 (fold change = 3.04, P < 0.05) were up-regulated, while the expression level of ENST00000422183 (fold change = 2.11, P < 0.001) was down-regulated in TB patients compared with the healthy control subjects. The expression levels of ENST00000568177 and ENST00000449589 showed no significant differences between TB patients and healthy control subjects. The scatter plots are shown in Fig. 4.

### Receiver operating characteristic (ROC) curve analysis

**Figure 5:**
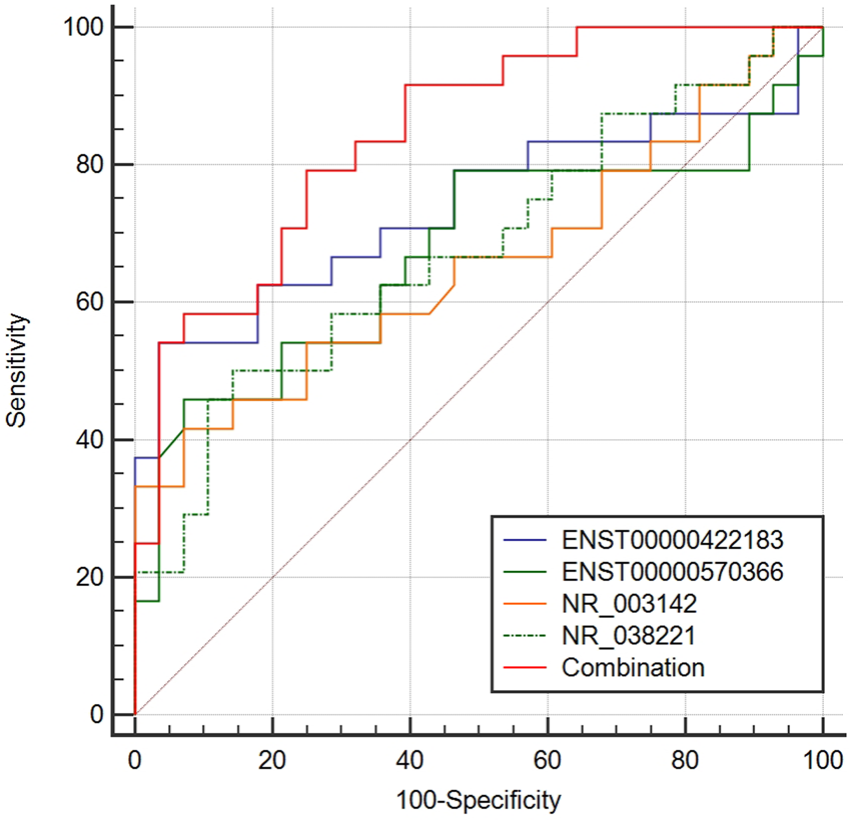
Receiver operating characteristic (ROC) curves analysis of the four lncRNAs. The AUC of the combination of four lncRNAs was 0.845 (95%CI, 0.742–0.949; P < 0.001, sensitivity = 79.2%, specificity = 75%). NR_038221 (0.677, 95%CI, 0.528–0.826; P = 0.029), NR_003142 (0.657, 95%CI, 0.503–0.811; P = 0.05), ENST00000570366 (0.672, 95%CI, 0.515–0.829; P = 0.034), and ENST00000422183 (0.738, 95%CI, 0.592–0.884; P = 0.003).

ROC curves were constructed to evaluate the diagnostic values of the differentially expressed lncRNAs. The area under the curve (AUC) value of the combination of the four lncRNAs was 0.845 (95%CI, 0.742–0.949; P < 0.001, sensitivity = 79.2%, specificity = 75%), which was much higher than the AUC values of NR_038221 (0.677, 95%CI, 0.528–0.826; P = 0.029), NR_003142 (0.657, 95%CI, 0.503–0.811; P = 0.05), ENST00000570366 (0.672, 95%CI, 0.515–0.829; P = 0.034), and ENST00000422183 (0.738, 95%CI, 0.592–0.884; P = 0.003) (Fig. 5).

### LncRNA-mRNA-miRNA network construction

**Figure 6:**
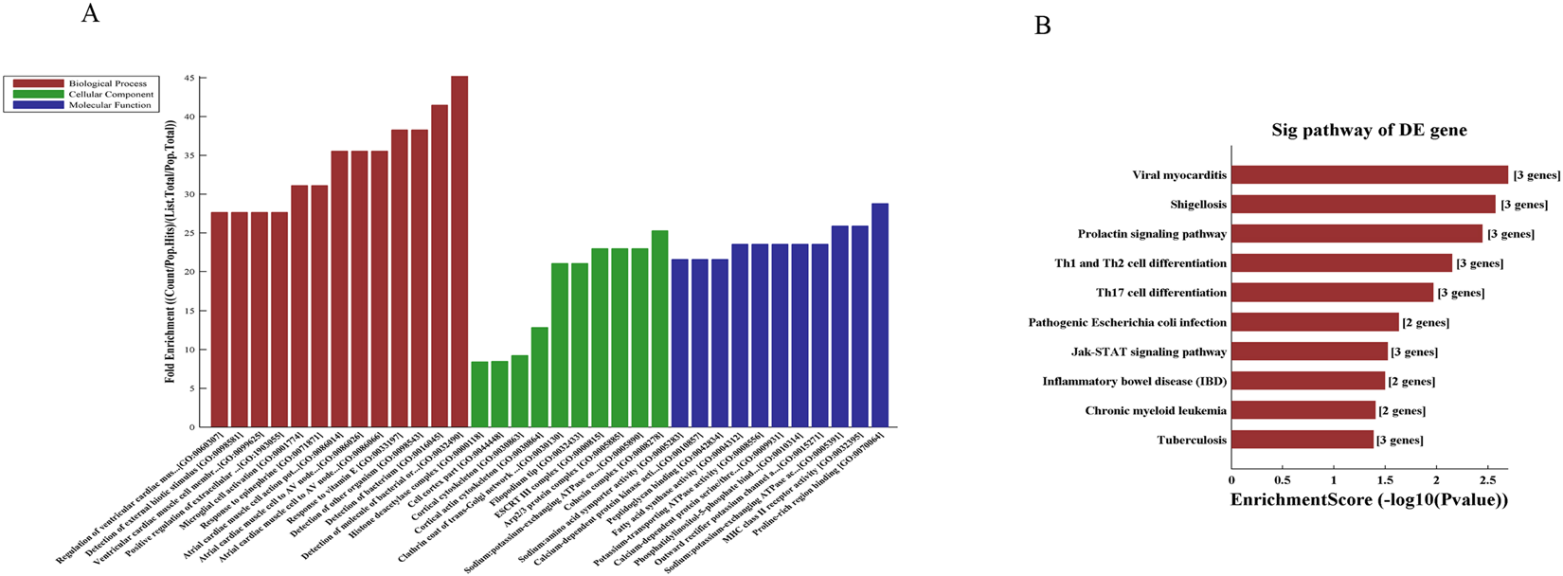
GO analysis of lncRNA-NR_038221 related to mRNAs according to the ceRNA analysis. (**A**) The GO items were enriched in regulation of transmembrane transport, detection of bacterium, MHC class II receptor activity, etc. (**B**) KEGG pathways enriched in Th1 and Th2 cell differentiation, tuberculosis and the Jak-STAT signalling pathway.

**Figure 7:**
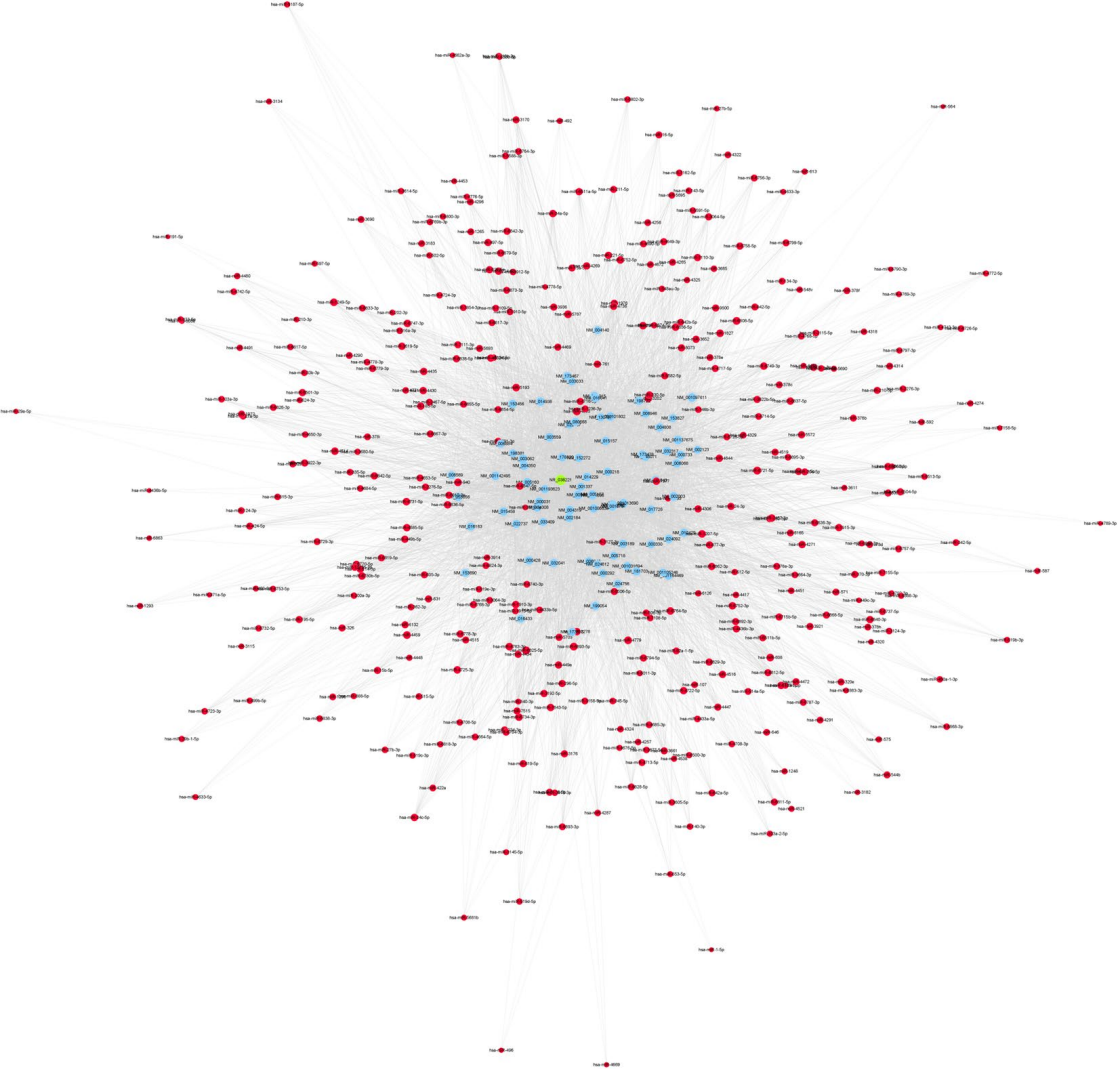
CeRNA analysis with lncRNA-NR_038221. 78 mRNAs and 368 miRNAs were associated with lncRNA-NR_038221. The blue node represents the coding gene, the red node represents miRNAs, and the green node represents the lncRNA-NR_038221.

Based on the qPCR results, we constructed a lncRNA-mRNA-miRNA ceRNA network. The results showed that NR_038221, NR_003142, and ENST00000570366 were correlated with mRNAs and miRNAs. A total of 85 mRNAs and 404 miRNAs were correlated with the four lncRNAs (Supplementary Table 3). GO and KEGG analysis were performed to predict the potential functions of the lncRNA-mRNA-miRNA networks, and lncRNA-NR_038221 was found to be the most related with TB, of which the GO items were enriched in the detection of molecules of bacterial origin, in the positive regulation of extracellular matrix organization and in the KEGG of JAK-STAT signalling pathway and TB (Fig. 6). Further ceRNA analysis results showed that 78 mRNAs and 368 miRNAs (Supplementary Table 3) were associated with lncRNA-NR_038221, where the potential target genes included TLR6, NOD2, and CD3E (Fig. 7).

## Discussion

TB is still one of the highest incidence and mortality diseases in the world. *MTB* can be transmitted through droplet nuclei produced by dried respiratory droplets. A droplet nucleus with a diameter of 1–5 µm contains 1–10 *MTB* that can exist in air for several hours and have the ability to enter the alveoli. On the other hand, respiratory droplets that are >100 μm in diameter will fall to the ground within 1 meter of origination and will enter the upper airway^22^. Bad habits, such as spitting, and a high population density in some areas might be the reasons for the high incidence of TB in China. Early and accurate diagnosis of TB is important for effective control and treatment of TB infection and to prevent TB from developing into MDR-TB^1^. However, commonly used methods, such as AFB and sputum culture, are not adequate for early and accurate diagnosis of TB because of the poor sensitivity and specificity for AFB and the long time required for a sputum culture test^23^. Therefore, it is important to find effective early diagnostic biomarkers for the prevention and treatment of TB.

We used microarray analysis to identify differentially expressed lncRNAs and mRNAs in the plasma of patients with TB. The results showed that 163 lncRNAs were up-regulated and 348 lncRNAs were down-regulated, while 127 mRNAs were up-regulated, and 284 mRNAs were down-regulated. GO analyses showed that the differentially expressed mRNAs were involved in regulation of alpha-beta T cell activation, T-helper 1 type immune response, positive/negative T cell selection, and cellular response to IFN-γ. KEGG results showed that the differentially expressed mRNAs were involved in the Jak-STAT signalling pathway and T cell receptor signalling pathway. These results suggest that the differentially expressed lncRNAs in TB patients might affect the activation of T cells and T helper cells, thus leading to abnormal immune functions. A previous study showed that alpha/beta T cells play important roles in the secretion of antigen-specific IFN-γ and can influence the immune response to *MTB* ^24^. MHC I- and MHC II-dependent immune responses play crucial roles in immunity against *MTB*, and CD4+ T cells and CD8+ T cells are associated with the formation of granulomas^25^. IFN-γ could limit the infection of *MTB*, while IL17 could induce IFN-γ secretion by CD4+ T cells^26^. Our microarray results also indicated that the functions of the aberrantly expressed lncRNAs were enriched in T cell regulation and related signalling pathways. In addition, it has been demonstrated that abnormal expression of lncRNAs in CD8+ T lymphocytes of TB patients can affect the cAMP signalling pathway, the calcium signalling pathway, the TGF-beta signalling pathway, and cytotoxicity mediated by natural killer cells^17^. Meanwhile, lncRNAs have also been demonstrated to be abnormally expressed in the CD4+ T cells of TB patients^19^. All these studies suggest that abnormal expression of lncRNAs is associated with TB and could affect the immune response by regulating CD4+ T and CD8+ T cells. We hypothesized that lncRNAs could modulate the immune response by influencing the activation and selection of T cells, thus affecting the progression of TB.

We also performed CNC analysis of lncRNAs and mRNAs. The results showed that the potential target genes of differentially expressed lncRNAs in TB mainly consist of IL6ST, IL18, BCL2, IL5, TLR6, and NOD2. *MTB* and its cellular components can be identified by Toll-like receptors (TLRs), thus activating the macrophage system, and then inducing immune responses, such as innate immunity and acquired immunity^27^. NOD2 is an important intracellular receptor for regulating the immune response to TB, which contributes to the inflammatory response and has been shown to be associated with the survival of macrophages after *MTB* infection^28^. The interleukin family can play an important role in the immune response to TB. *MTB* infection can cause increases in IL-17, IL-6 and TNF expression levels^29^. IL18, secreted by macrophages after *MTB* infection, has been shown to be related to the activation of T cells^30^. The results of CNC analysis showed that differentially expressed lncRNAs in patients with TB could affect the pathological process of TB by regulating related receptors, such as TLR6, NOD2, Bcl2, IL-5, IL-18, IL6ST, and other interleukin groups.

Several studies have shown that lncRNAs could serve as molecular biomarkers for diagnosis and prognosis of diseases. For example, the expression level of lncRNA UCA1 in plasma is important for early diagnosis of gastric cancer^31^. Not only can the abnormal expression of lncRNAs influence the pathological process of TB but so can polymorphisms of lncRNAs associated with the susceptibility to TB, suggesting that lncRNAs have profound effects on the pathogenesis of TB^32^. The complexity of the pathological process of TB suggests that a single marker may not be suitable for the diagnosis of TB. Our results showed that four lncRNAs were abnormally expressed between TB patients and healthy controls. Among them, NR_038221 (fold, change = 3.79, P < 0.01), NR_003142 (fold, change = 1.69, P < 0.05), and ENST00000570366 (fold, change = 3.04, P < 0.05) were up-regulated, while ENST00000422183 (fold, change = 2.11, P < 0.001) was down-regulated. We used the ROC curve to assess the diagnostic value of these four lncRNAs for TB. The AUC values of the four lncRNAs were as follows: NR_038221 (0.677), NR_003142 (0.657), ENST00000570366 (0.672), and ENST00000422183 (0.738). We used the logistic regression method to assess the four lncRNAs as a model to diagnosis TB, and the AUC value was found to be 0.845 with a sensitivity = 79.2% and specificity = 75%. The results indicated that a model consisting of the combination of four lncRNAs performed better than a single marker to diagnose TB.

ceRNA analyses have been shown to predict the potential relationships between non-coding RNAs, including circRNAs and lncRNAs^33,34^. We performed ceRNA analysis of NR_038221, NR_003142, ENST00000570366, and ENST00000422183, which were differentially expressed as shown by qPCR. A total of 85 mRNAs and 404 miRNAs were predicted and related with the four differentially expressed lncRNAs. Combined with the CNC analysis results, we further performed GO and KEGG analyses of these mRNAs. The results showed that NR_038221 was the most significantly associated with TB. The ceRNA analysis showed that NR_038221 interacted with 78 mRNAs and 368 miRNAs. The GO functions were enriched in the regulation of transmembrane transport and the detection of bacteria. The potential target genes were TLR6, NOD2, HLA-DQB and IL6ST, suggesting that NR_038221 might affect the pathological process during TB infection by influencing signal transduction between immune cells and the intracellular signalling pathway. Our previous study verified six miRNAs as potential biomarkers for pulmonary TB, including hsa-miR-378^6^, of which the mature sequence hsa-miR-378a-3p was among the 368 miRNAs that associated with NR_038221, indicating that NR_038221 and hsa-miR-378a-3p might play a similar function during the pathological process of pulmonary TB.

In summary, we identified four differentially expressed lncRNAs in plasma of TB patients that may influence the activation of T cells. These lncRNAs could affect signal transduction between cells, thus influencing the pathological process of TB. We propose that these four lncRNAs could serve as potential biomarkers for the diagnosis of TB. The combined diagnostic model had favourable a diagnostic effect and provides a new basis for the early diagnosis of TB.

## Materials and Methods

### Patients and control subjects

Primary pulmonary TB patients were diagnosed using the criteria described by the Chinese Ministry of Health (China). Patients with autoimmune diseases, infection diseases, cancer and other diseases were excluded in this study. A total of 114 blood samples of active TB (88 males and 26 females) ageing 18–65 years (mean age 40.27 ± 16.79) were collected from the Six Hospital of Shaoxing (China) between January 2015 and December 2016. Healthy control subjects were recruited from the Zhejiang Hospital (Hangzhou, Zhejiang, China), and 105 blood samples (73 males and 32 females) from patients aged 18–65 years (mean age 36.45 ± 12.24) were collected. Fasting blood samples were collected in EDTA anti-coagulation tubes and then centrifuged (3000 rpm) at 4 °C for 10 min to separate plasma. Plasma samples were dispensed in RNase free tubes and stored at −80 °C. This study was approved by the Ethics Committee of the Department of Medicine, Zhejiang University (China). Written informed consent was obtained from all TB patients and healthy control subjects enrolled in this study. All methods we used in this study were performed in accordance with the relevant guidelines and regulations.

### Microarray analysis of lncRNAs and mRNAs

A total of 33 TB patients and 11 healthy control subjects were analysed using Arraystar Human LncRNA Microarray V3.0 (Array-Star, Inc., Rockville, MD, USA) in this study. All subjects were divided into three biological repeats. In brief, TRIzol® reagent (Invitrogen life technologies) was used to isolate total RNA from the plasma of TB patients and healthy control subjects. Total RNA was purified using an RNasey Mini Kit (Qiagen p/n 74104), and RNA quantification and quality were determined by NanoDrop ND-1000. RNA samples were labelled by the Quick Amp Labeling Kit, One-Color (Agilent p/n 5190–0442), and the labelled cRNA were purified and then hybridized onto the microarrays, which contained 30586 lncRNA and 26109 coding transcripts probes, and then scanned by Agilent Microarray Scanner (Agilent p/n G2565BA). Agilent Feature Extraction software (version 11.0.1.1) was used to analyse the array images. Raw signal intensities were normalized in quantile method by GeneSpring GX v11.5.1 (Agilent Technologies).

### GO and KEGG pathway analysis

The GO analysis (http://www.geneontology.org/) was used to describe gene functions. It classified functions along three aspects: molecular function, cellular component, and biological process. Fisher’s exact test was used to detect if there were enrichment between the GO category and differentially expressed mRNAs. The GO terms were considered to be significantly enriched if P-value was < = 0.05. Pathway analysis is a functional analysis of mapping genes to KEGG pathways. The differentially expressed mRNAs were involved with the biological pathways if the P-value was < = 0.05.

### Construction of the coding-non-coding gene co-expression (CNC) network

The CNC network was constructed to study the relationship between differentially expressed lncRNAs and mRNAs, as described in previous studies^35,36^. The steps to construct the CNC network were as follows: (1) Genes associated with the GO enrichments and KEGG pathways according to the GO and pathway analyses were selected, and the median value was calculated if the coding genes have several transcripts. (2) Pearson correlation coefficient (PCC) was calculated between lncRNAs and coding genes, and the correlation was considered to be meaningful if the PCC > = 0.85. (3) Cytoscape (v3.4.0) was used to construct the co-expression network.

### RNA extraction and quantitative real-time PCR (qPCR)

Total RNA was extracted from 600 μl of plasma of each subject using TRIzol® reagent (Invitrogen life technologies), and the quantification and quality of total RNA were measured with NanoDrop 2000 (Thermo Scientific). In total, 1.5-μg RNA samples were reverse transcribed into cDNA using PrimeScript ™ RT Master Mix ((Perfect Real Time, Takara), and qPCR was performed with SYBR ® Premix Ex Taq™ (Tli RNaseH Plus, Takara) using LightCycler 480 II (Roche) according to the manufacturer’s protocol. The qPCR reaction started with initial denaturation at 95 °C for 30 s, followed by 40 cycles at 95 °C for 5 s, 62 °C for 30 s. All quantitative PCR reactions were performed in triplicate and 18 S RNA was used as internal control. Relative lncRNAs levels were calculated by the 2^−ΔΔCt^ method, and melt curve analysis was performed to confirm non-specific amplification. The primer sequences of lncRNAs are listed in Supplementary Table 2.

### Prediction of interaction between lncRNAs, miRNAs, and mRNAs

The ceRNA analysis was performed to predict the core-interaction between differentially expressed lncRNAs, miRNAs, and mRNAs according to previous studies^37–39^. In brief, the overlapped miRNAs predicted by differentially expressed lncRNAs, which were verified by qPCR as downstream targets, and mRNAs based on the microarray results as upstream regulators were enriched, and then the lncRNAs-mRNAs-miRNAs ceRNA network was constructed.

### Statistical analysis

All results were analysed with SPSS 18.0 (SPSS, Chicago, IL). The experimental results were presented as the mean ± SD, and the P value of <0.05 was considered as statistically significant. The non-parametric Mann–Whitney U test was used to compare continuous variables such as the qPCR results. The chi-square test was used for qualitative variables such as the gender between TB and healthy control groups. ROC curves were used to evaluate the diagnostic values of the lncRNAs biomarkers and were performed with MedCalc statistical software.

## Electronic supplementary material

Supplementary information

Supplementary table 3
