# Supplementary material for: Screening and identification of lncRNAs as potential biomarkers for pulmonary tuberculosis
Source: Sci Rep. 2017 Dec 1;7:16751. doi: 10.1038/s41598-017-17146-y (PMC5711916; doi:10.1038/s41598-017-17146-y)
Supplement: Supplementary file 1 — Supplementary information [file 41598_2017_17146_MOESM1_ESM.pdf]

Screening and identification of lncRNAs as potential biomarkers for pulmonary tuberculosis

Zhong-liang Chen[1] , Li-Liang Wei[2] , Li-Ying Shi[3] , Meng Li[3], Ting-Ting Jiang[4], Jing Chen[1], Chang-Ming Liu[1] , Su Yang[1], Hui-hui Tu[1], Yu-ting Hu[4], Lin Gan[4], Lian-Gen Mao[1] , Chong Wang[1] & Ji-Cheng Li[1,4]*

[1] Institute of Cell Biology, Zhejiang University, Hangzhou 310058, P.R. China. [2] Department of Respiratory Medicine, The Sixth Hospital of Shaoxing, Shaoxing 312000, P.R. China. [3] Department of Clinical Laboratory, Zhejiang Hospital, Hangzhou 310013, P.R. China. [4]South China University of Technology School of Medicine, Guangzhou 510006, P.R. China. Correspondence and requests for materials should be addressed to J.-C.L. (email: lijichen@zju.edu.cn)

Supplementary Table 1: GO and KEGG categories

1. Potentially associated with the regulation and differentiation of T cell;GO:0045060 negative thymic T cell selection

GO:0043383	negative T cell selection
GO:0045058	T cell selection
GO:0046635	positive regulation of alpha-beta T cell activation
GO:0046634	regulation of alpha-beta T cell activation
GO:0046631	alpha-beta T cell activation
GO:0050870	positive regulation of T cell activation
GO:0045639	positive regulation of myeloid cell differentiation
hsa04660	T cell receptor signaling pathway - Homo sapiens (human)

2. Antigen presentation and transmembrane signal transduction GO:0042611 MHC protein complex

GO:0002504	antigen processing and presentation of peptide or polysaccharide antigen via MHC class II
GO:0042613	MHC class II protein complex
GO:0016045	detection of bacterium
GO:0051043	regulation of membrane protein ectodomain proteolysis

3. Intracellular signal transduction pathway.GO:0007243 intracellular protein kinase cascade

GO:0007263	nitric oxide mediated signal transduction
GO:0071346	cellular response to interferon-gamma
hsa04630	Jak-STAT signaling pathway - Homo sapiens (human)

Supplementary Table 2: lncRNA and primer

LncRNAs selected for further verification	
ENST00000422183-F	AGTCCTAAAGAGCAAGCCTA
ENST00000422183-R	CCGACTCTCCATACCCAA
ENST00000568177-F	ATCGCCATTCTCCAGTCT
ENST00000568177-R	TAATCCTCCTCAGCACAGA
NR_003142-F	CGTCACCCGAAAAGCGACT
NR_003142-R	CGCGGACGTTTATAGCCACT
NR_038221-F	GGCAGCAGGAGAAGAATC
NR_038221-R	GAAGCAGTCAGAGGATGTAA
ENST00000449589-F	TGGATAGCACCTTATGGACAG
ENST00000449589-R	CCTCATCATTCTAGCACTCAAG
ENST00000570366-F	CTGATTCCCGACATACAGCA
ENST00000570366-R	TAAATCATGGCTCTAAGACGAC
18S-F	CGGCTACCACATCCAAGGAA
18S-R	GCTGGAATTACCGCGGCT
All other lncRNAs	
ENST00000537024-F	AATGTTCAGCCCACAAGAGC
ENST00000537024-R	TCATGTATTTTCACTGTCGGTCA
TCONS_00023029-1F	GCTCAGCGCGACACCACGACCAC
TCONS_00023029-1R	GGGTCCTGGTTCCTTTGACTATCGCTT
TCONS_00023029-2F	AGCACAGCCCCTCTCTCCGT
TCONS_00023029-2R	TCCTGGTTCCTTTGACTATCGCTT
ENST00000432162-F	ATTTCCCCAAGAGCATACCAC
ENST00000432162-R	ACAAAGGCAGAACATCCCAT
TCONS_00006918-F	AACTGTTCTTAGGTAGCTCGT
TCONS_00006918-R	GCAATAGATATAGTACCGCAAG
uc010mmq.1-F	CCTGGTCTGTTCTGTATTGT
uc010mmq.1-R	GCCTCTACTTCGGTCTCT
ENST00000474667-F	TTAGGAGAGGCTGCGAAT
ENST00000474667-R	GAGAGGCTGGAGTGTTAAG
TCONS_00029621-F	AGGACACAGCAAGAAGGT
TCONS_00029621-R	CAGCACTGACAGATCAACA
ENST00000580993-F	CGCAGTCACTAGAAGTTCAT
ENST00000580993-R	CGTGTTACTCAGCTCCATT
ENST00000457658-F	TTTGTCTTACTTGGGACGTT
ENST00000457658-R	CGAGCTTACACGGACTCCA
ENST00000442823-F	GCCTTACCGTATAACTGACT
ENST00000442823-R	TTACAGACTCCAGCAGGTA
ENST00000427373-F	TCAGCAGGAGGAGATAAGG
ENST00000427373-R	GCGAGCAAGTGGTTAGAA
uc004dvh.2F	TGGAATAGCAGGCATAACC

uc004dvh.2-R	CATTAGTAACAGGAAGAGGAAG
ENST00000443799-F	ATTATCTTAACTGAGGCACCCAA
ENST00000443799-R	CAAGACAAGAATCCGCCAT
ENST00000563833-F	GCATACAGACAGACGGACACA
ENST00000563833-R	GGCTATGGACCGCATGACTCT
TCONS_00027244-F	CCAGCAATACATCTCAAGCACT
TCONS_00027244-R	TTTGTGACATATTGCTATACCCAGA
ENST00000414345-F	TTCAAGCACTATCCGATTCA
ENST00000414345-R	CCAGAAGAAGAAGGCAACA
TCONS_00019576-F	ATCACTCTGCCACCATCT
TCONS_00019576-R	TGTTTACAATTCACGCTCTG
TCONS_00001219-F	ACCTCTCACATGGACCTT
TCONS_00001219-R	CACAGCCTAATAGCACAGA
ENST00000421297-F	TGAGGACATAAGGACAGATG
ENST00000421297-R	AAGAAGAAGCAAGTTGATGG
ENST00000580595-F	GTCCTGAGTTCTCTTCCTG
ENST00000580595-R	ACCGTTACCATTACTGAGTT
ENST00000521863-F	CGTCGCTGTTATCTCATTGTCT
ENST00000521863-R	ATCAAGGTCCGCTGTGAAC
TCONS_00016476-F	TTGAGGCTTCCAGGCACT
TCONS_00016476-R	CCACACTGAGGAGATTGTTCT
TCONS_00025483-F	CCTTACCACACCGCTCTTC
TCONS_00025483-R	GGCTCTCTTGGGTCTAACTG
TCONS_00001406-F	GAGGAAGGAGGAGTGTGAAC
TCONS_00001406-R	CGAGGAGGAGGATGAAGGT

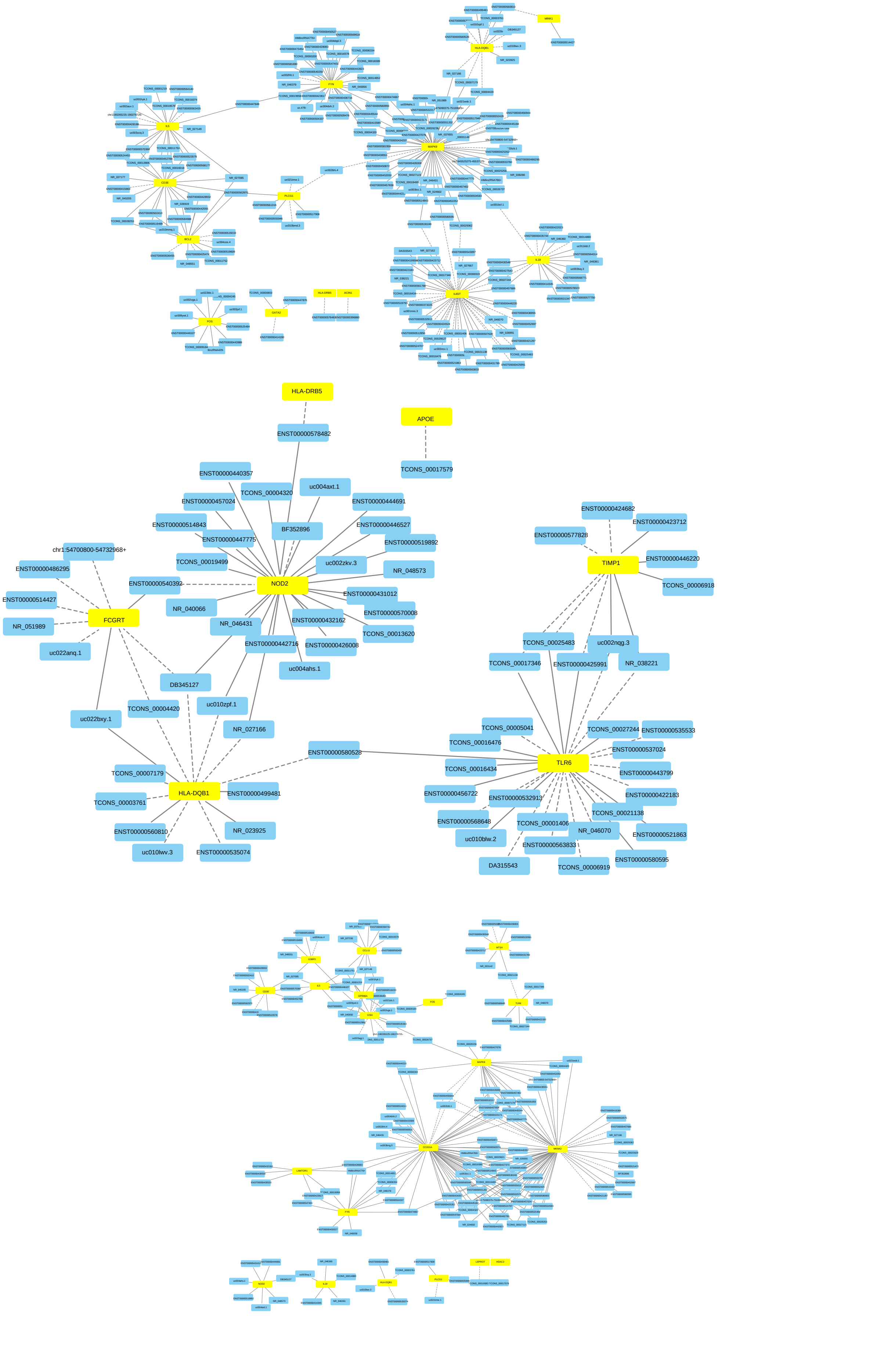

Supplementary Figure S1. The complete results of the CNC network. CNC analysis results according to three categories: (i) Potentially associated with the regulation and differentiation of T cell; (ii) Antigen presentation and transmembrane signal transduction; and (iii) The intracellular signal transduction pathway.
